# Neuraxial and Regional Anesthesia in a Patient With Amyotrophic Lateral Sclerosis: A Case Report

**DOI:** 10.7759/cureus.37364

**Published:** 2023-04-10

**Authors:** Alexander G Samworth, Kenneth Miller, Muin Haswah, Luminita Tureanu, Jessica Weeks

**Affiliations:** 1 Regional Anesthesiology, Northwestern University Feinberg School of Medicine, Chicago, USA; 2 Regional Anesthesiology, University of California San Diego, San Diego, USA; 3 Regional Anesthesiology, North Star Anesthesia, Irving, USA

**Keywords:** adductor canal block, primary total knee arthroplasty, spinal anesthesia, amyotrophic lateral sclerosis, regional anesthesiology

## Abstract

Patients with amyotrophic lateral sclerosis (ALS) who undergo lower extremity joint arthroplasty are rarely encountered. Patients with ALS are at an increased risk for perioperative anesthetic complications. Anesthetic techniques, regional or general, present different risks to patients with ALS. The historical concern of worsening pre-existing neurologic symptoms with regional anesthesia is being re-examined in light of emerging evidence supporting its use in patients with ALS. Here, we present the successful perioperative management of a patient with severe bulbar ALS undergoing total knee arthroplasty. Despite his advanced bulbar symptoms, he was independently ambulatory with severe knee pain related to osteoarthritis. During multidisciplinary planning with the patient and his wife, it became clear that his primary perioperative concern was avoiding intubation, prolonged ventilation, and tracheostomy placement. With this in mind, we planned for a neuraxial anesthetic without intraoperative sedation, a postoperative adductor canal peripheral nerve block, and multimodal non-opioid analgesia. There were no perioperative complications. At the six-week follow-up, he experienced improved ambulation and showed no signs of worsened ALS symptoms.

## Introduction

Amyotrophic lateral sclerosis (ALS), commonly called Lou Gehrig's disease, is the most common motor neuron disease, with an incidence of 1-2.6 cases per 100,000 persons annually [1]. Clinical signs and symptoms are variable with limb-onset (extremity weakness) and bulbar-onset (difficulty chewing and swallowing), among others, being described [2]. Diagnosis is primarily one of exclusion and is based on the Gold Coast criteria [3]. There is no cure, and treatment focuses on symptomatic management. Progression of ALS causes significant impairment of respiratory function and airway clearance, often necessitating support devices such as noninvasive positive pressure ventilation. Patients with ALS are at an increased risk for perioperative complications and anesthesiologists caring for them must be prepared to manage their complex medical conditions [4].

Anesthesiologists typically encounter patients with ALS for procedures related to their disease's progression such as gastrostomy and tracheostomy tube placement [4]. However, patients with ALS undergoing lower extremity joint replacement are rarely encountered, and no definitive guidelines exist for their perioperative care [5]. General anesthesia may worsen respiratory function and lead to prolonged invasive mechanical ventilation and other pulmonary complications [4]. Alternatively, regional anesthesia is traditionally avoided in patients with ALS, as it may worsen pre-existing neurologic symptoms [6,7]. However, there is growing support and evidence for the safe use of regional anesthetic techniques in patients with ALS [5,8,9].

Here, we present the successful perioperative management of a patient with severe bulbar ALS undergoing right total knee arthroplasty (TKA) utilizing neuraxial anesthesia, regional analgesia, and non-opioid analgesics. Written HIPAA (The Health Insurance Portability and Accountability Act of 1996) authorization was obtained to submit this case report from the patient's power of attorney. This document contains no identifiable patient information and is exempt from IRB review requirements as per institutional policy. This manuscript adheres to the applicable EQUATOR guideline [10].

## Case presentation

An 80-year-old man, American Society of Anesthesiologists (ASA) physical status IV, with severe bulbar ALS was scheduled for right TKA. His preoperative vital signs, laboratory tests, and physical exam were normal. His ALS symptoms included tongue atrophy, independent ambulation with an assistive device, sialorrhea, and head ptosis. He required non-invasive positive pressure ventilation at night and received botulinum toxin injections regularly to reduce oral secretions. His bronchial hygiene routine involved self-suctioning, mechanical insufflation-exsufflation, and albuterol nebulizer treatments. He received nutrition through a gastric tube. Despite his severe ALS, his ambulation was primarily limited by knee pain from osteoarthritis, and this was negatively impacting his quality of life. After extensive counseling from his medical care team, he elected to proceed with TKA.

Prior to surgery, a multidisciplinary team, including orthopedic surgery, anesthesiology, and pulmonology, met to discuss the patient's anticipated surgical challenges: increased risk of aspiration, prolonged ventilation, and potential for worsening neurologic symptoms. The patient expressed that he was most concerned with avoiding perioperative tracheostomy placement. With these concerns in mind, the team recommended proceeding with a modified version of our institution’s established clinical pathway for TKA. The routine pathway includes preoperative oral analgesics, a spinal anesthetic with intraoperative sedation, periarticular joint infiltration, and multimodal postoperative analgesia, including an adductor canal (AC) peripheral nerve block and postoperative analgesics as needed.

Our patient’s anesthetic included a single-shot spinal at the lumbar 3-4 interspace in the sitting position using the anatomic landmark technique. A 25-gauge 8.9 cm pencil point spinal needle was inserted through a 20-gauge 3.2 cm introducer needle (B. Braun Medical Inc., Bethlehem, PA). A total of 2.2 mL of 0.5% isobaric bupivacaine was administered into the intrathecal space. He received no intraoperative sedation to reduce the risk of respiratory compromise and the need for ventilatory support. To reduce the risk of aspiration, the patient received famotidine in the preoperative period, was positioned with 30 degrees of head elevation in the operating room, and his gastrostomy tube was under suction throughout the case. The patient was given a Yankauer suction device to self-manage his oral secretions. Prior to closure, the surgical team performed a periarticular joint infiltration with epinephrine, ropivacaine, ketorolac, clonidine, and morphine. Postoperatively, the anesthesiology team performed an ultrasound-guided AC peripheral nerve block using 10 mL of 0.25% bupivacaine. The AC block was performed in the supine position using an in-plane technique with a 21-gauge 100 mm stimulating block needle (Pajunk, Medizintechnologie, Geisingen, Germany. Ultrasound: 13-6 MHz probe, Fujifilm Sonosite, Bothell, WA).

Following discharge from PACU, he was admitted to the surgical intensive care unit for close monitoring. His postoperative analgesic regimen included acetaminophen and ketorolac. He did not require any opioids. He was transferred to the floor on postoperative day one and discharged home on postoperative day two. At his two-week follow-up appointment, he was ambulating with the assistance of a walker. At six weeks, he was able to ambulate with a cane and there was no identified progression of his neurologic symptoms. The patient and his wife expressed gratitude to the entire team for the care he received.

## Discussion

ALS is a rapidly progressive neurodegenerative disease affecting upper and lower motor neurons. ALS often presents in the fifth and sixth decades of life with muscle weakness, atrophy, fasciculations, and spasticity [2]. Progression is rapid and patients typically die of respiratory failure within three years of symptom onset [11]. Patients with ALS present unique challenges to the anesthesiologist [4]. Anesthetic concerns in these patients include gastric aspiration, postoperative ventilatory support, autonomic instability, and an unpredictable increased sensitivity to opioids, sedatives/hypnotics, and non-depolarizing neuromuscular blocking agents [5]. In addition to these considerations, previous case reports raised concern for the worsening of pre-existing neurologic symptoms with the use of regional anesthesia [6,7]. The “double-crush” phenomenon proposed that a nerve with a pre-existing compressive lesion is more susceptible to subsequent compressive injury [12]. The theory is extrapolated to patients with motor neuron disease such that the ‘first crush’ is their underlying neurologic disorder and the ‘second crush’ occurs when exposed to mechanical, ischemic, or toxic insults commonly associated with regional anesthetic techniques.

However, recent evidence supports alternative explanations for these prior observations. For one, surgical stress has been associated with postoperative inflammatory neuropathy irrespective of the anesthetic technique [13]. Similarly, surgical stress is suggested to be an evolving new risk factor in the diagnosis and progression of ALS [14]. Although previous reports associated worsening neurologic symptoms with regional techniques, other factors, including positioning, body habitus, and local anesthetic concentration, likely played a role, and isolating the direct impact of the anesthetic technique on the neurological outcome is difficult [5,8].

An increasing number of case reports have demonstrated the use of regional anesthetic techniques in patients with ALS without worsening their neurologic symptoms. Neuraxial anesthesia is the most well-documented. Reports of its safe use include patients undergoing cesarean delivery [15], total hip arthroplasty [5], and repair of a transtrochanteric fracture of the femur [16]. None of these patients experienced new or worsening neurologic symptoms. A case series of 139 patients with pre-existing CNS disorders, including five patients with ALS, all received neuraxial anesthesia, none of whom had worsening neurologic symptoms [8].

There are fewer case reports evaluating the use of peripheral nerve blocks in ALS patients. One group reported using a lumbar plexus catheter and single-shot sciatic nerve block for a femoral neck fracture repair in a patient with ALS. This patient experienced no perioperative complications or progression of neurologic disease at three months follow-up [9].

Despite these reports, no consensus on the use of regional anesthesia in patients with ALS exists [5]. In formulating the anesthetic plan for our patient, we had a multidisciplinary discussion involving anesthesiology, orthopedic surgery, pulmonology, the patient, and his wife. The factor most important to our patient was avoiding airway manipulation and the need for invasive ventilation. Based on current evidence, we believed a regional anesthetic would best align with his care goals while being a safe option [8]. With his severe bulbar symptoms, we opted to forgo preoperative anxiolysis and intraoperative sedation to minimize changes to his carbon dioxide ventilatory response curve. This required a motivated patient and a cooperative surgical team. Similarly, the decision to perform a postoperative peripheral nerve block and utilize multimodal non-opioid analgesics proved an effective method to eliminate the use of opioids in the perioperative period.

## Conclusions

Mounting evidence supports the safe use of regional anesthetic techniques in patients with ALS. In this report, combined neuraxial and peripheral nerve block techniques were used in a patient with severe ALS undergoing TKA. No postoperative worsening of his neurologic symptoms was observed. As ever, a thorough risk, benefit, and alternatives discussion is essential to the care of patients with pre-existing motor neuron disease to ensure that their anesthetic plan aligns with their goals of care. Further research is required to clarify the optimal anesthetic technique for patients with ALS undergoing lower extremity joint replacement.
